# Beef Tea

**Published:** 1902-12-20

**Authors:** 


### Editor's Letter-Box.

[Our Correspondents are reminded that prolixity is a great bar to publication, and that brevity of style and conciseness of statement greatly facilitate early insertion.]

### BEEF TEA.

The Matron of the Union Workhouse, Newbury, writes:—Having seen a letter in November's issue of The Hospital *re* "Beef Tea *verses* Bovril," I can fully endorse the statements therein, having used Bovril in this Workhouse Infirmary for the last eight months.

I find a saving, due to Bovril, of over 50 per cent. in cost, exclusive of fuel and trouble in preparation.

It is prepared instantaneously, and gives satisfaction to all the patients.

